# CHANGES IN FUNCTIONAL STATUS AMONG US ADULTS IN LATE MID-LIFE FROM THE HEALTH AND RETIREMENT STUDY 2004–2016

**DOI:** 10.1093/geroni/igac059.1155

**Published:** 2022-12-20

**Authors:** Adit Doza, Elham Mahmoudi, Wassim Tarraf

**Affiliations:** University of Michigan, Ann Arbor, Michigan, United States; University of Michigan, Ann Arbor, Michigan, United States; Wayne State University, Detroit, Michigan, United States

## Abstract

Reducing racial/ethnic disparities in health and functioning among older adults are salient key goals in the U.S. health policy. This study examined whether and how the functioning have progressed between White and minority populations in late midlife in recent years. We analyzed the Health and Retirement Study (HRS) among adults ages 45-64 years across two time periods (2004-2010 and 2010-2016). Using generalized linear regression, we modeled changes in activities of daily living (ADL) and changes in instrumental activities of daily living (IADL) as a function of race/ethnicity and sequentially adjusted for period specific risk factors including sociodemographic factors, health insurance, health behaviors and social networks. Oaxaca-Blinder Regression Decomposition (OBRD) techniques are used to assess the contributions of these factors to the observed trends in ADL/IADL. We find changes in ADL is significantly higher for Blacks (Odds Ratios of 1.23, CI 1.01-1.48) and US-born Hispanics (Odd Ratios of 1.54, CI 1.12-2.12) after adjusting for all the risk factors in the second period. About two-thirds of the disparity in functional status of adults is explained in our OBRD model and more than 70% of these explained differences among minorities is related to socio-demographic factors. This probably occurred due to the differences in income and net wealth which began to widen during the time of 2008 financial crisis. Although recently there is a decline in disability trends for older adults in U.S., our findings point to an increase in racial/ethnic disparities in functional status for adults in late midlife.

